# Tuberculosis and neuromyelitis optica spectrum disorder: A conundrum

**DOI:** 10.22336/rjo.2022.51

**Published:** 2022

**Authors:** Rolli Khurana, Mohini Agrawal, Raman Mehta, Sandeep Shankar

**Affiliations:** * Department of Ophthalmology, Military Hospital, Ahmedabad, India; ** Department of Ophthalmology, Command Hospital, Pune, India; *** Department of Pediatric Ophthalmology, Strabismology and Neuroophthalmology, Dr. Shroff’s Charity Eye Hospital, New Delhi, India; **** Department of Ophthalmology, Armed Forces Medical College, Pune, India

**Keywords:** tuberculosis, neuromyelitis optica, optic neuritis, paediatric, NMOSD

## Abstract

**Purpose:** Infectious atypical optic neuritis (AON), like tubercular, is a vision threatening condition with phenotypic overlap with Neuromyelitis Optica Spectrum Disorder (NMOSD). The overlapping neurological manifestations and negative AQP4-Ab-assay make it difficult to discover the primary cause of neuritis.

**Case presentation:** We report two paediatric cases with NMOSD that did not fulfil the diagnostic criteria. Moreover, associated undiagnosed tuberculosis at the time of presentation and negative AQP4-Ab clouded the diagnosis and delayed the treatment. The first case was initially diagnosed with infectious optic neuropathy. By the time steroids were started, optic atrophy had already set in. The second case had optic neuritis, LETM, and intracranial-tuberculomas with no signs of pulmonary-tuberculosis with negative CSF-analysis. So, systemic steroids were started promptly. The history of LETM in both cases raised the suspicion of NMOSD.

**Conclusion:** The importance of accurate clinical diagnosis and early intervention in cases of AON was emphasized in a limited resource country, that could potentially result in salutary visual outcomes, especially in the paediatric age group

**Abbreviations:** AON = atypical optic neuritis, TB = tuberculosis, NMOSD = neuromyelitis optica spectrum disorder, LETM = longitudinal extensive transverse myelitis, AQP4-Ab = anti-Aquaporin-4 Antibodies, RE = right eye, LE = left eye, MRI = magnetic resonance imaging, CSF = cerebrospinal fluid, ATT = anti-tubercular treatment, DOV = diminution of vision

## Introduction

Neuromyelitis optica is a rare autoimmune disease that specifically causes inflammation of the spine, as well as the optic nerve [**1**]. Neuromyelitis optica spectrum disorder (NMOSD) is the name given to conditions that do not meet the criteria for diagnosis of NMO, have seropositivity to Anti-Aquaporin-4 Antibodies (AQP4-Ab) but present with this combination of optic neuritis and transverse myelitis [**2**]. Infectious atypical optic neuritis (AON) like tubercular and neurosyphilis are also potential vision-threatening conditions with occasional phenotypic overlap with NMOSD. Both NMOSD and AON are rarer in children. The varied and non-specific symptoms often make it difficult to differentiate NMOSD from AON.

Here, we reported two paediatric cases with NMOSD that did not fulfil the diagnostic criteria. Associated undiagnosed tuberculosis at the time of presentation and negative AQP4-Ab clouded the diagnosis and delayed treatment in one of these cases. This emphasized the importance of accurate clinical diagnosis and early intervention. 

## CASE 1

A fifteen-year-old male presented with poor vision in the left eye (LE) for seven months and the right eye (RE) for six months. Vision was light perception (PL) with accurate projection of rays in both eyes. On ophthalmoscopy, bilateral diffuse optic disc pallor was noted. On retrospective evaluation, he reported an episode of fever, cough, followed by transient weakness in both lower limbs and urinary retention 10 days prior to involvement of LE. 

**Fig. 1:**
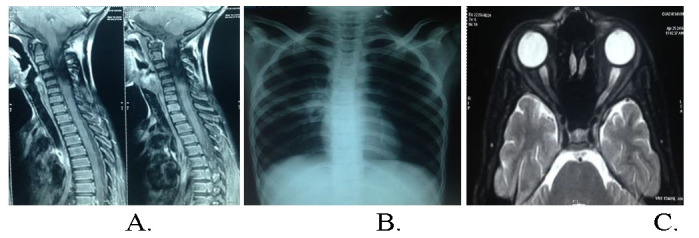
Radiological imaging of spinal cord and lung of Case 1 **A.** MRI of the spine suggestive of longitudinally extensive transverse myelitis; **B.** Chest X-Ray showing Koch’s lesions with cavitation in the right mid-zone, which was suspected to be tubercular; **C.** T2 Fat suppressed axial MRI of the orbits revealing hyperintensity in bilateral optic nerves

His MRI of the spine at the time of limb weakness (**Fig. 1A**) was suggestive of longitudinally extensive transverse myelitis (LETM). AQP4-Ab, thyroid dysfunction, B12 levels, viral markers and cerebrospinal fluid (CSF) analysis were negative. Koch’s lesions with cavitation on chest X-ray were suspicious of tuberculosis (**Fig. 1B**). He was started on anti-tubercular treatment (ATT). Within 10 days of commencement of ATT, he developed diminution of vision (DOV) in LE and was suspected of ethambutol toxicity. He was switched to modified ATT. 

At that point, immunosuppressants were not administered considering tubercular optic neuropathy. After one month, he developed rapidly progressive DOV in RE as well. Contrast enhanced MRI orbit and brain (**Fig. 1C**) revealed bilateral optic nerve enhancement up to the chiasma. Due to poor response to ATT and sequential ON, he was treated as a suspected case of NMOSD. Oral steroids 1 mg/ kg/ day and oral azathioprine 25 mg/ day were started. By that time, he developed optic atrophy in LE.

## CASE 2

**Fig. 2:**
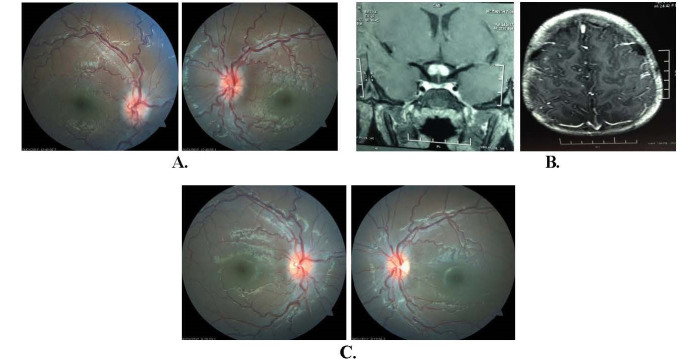
Fundus photography and radiological imaging of case 2 **A.** Fundus Photograph of Case 2 showing bilateral disc oedema with arteriolar attenuation and venous dilatation on Day 1; **B.** Contrast enhanced MRI of brain showing multiple enhancing nodular lesions in bilateral cerebral hemispheres at grey white matter interface suggestive of inflammatory tuberculomas; **C.** Fundus photograph showing improvement in bilateral optic neuritis on Day 3 of treatment with parenteral methylprednisolone under cover of anti-tubercular treatment

An eight-year-old boy presented with rapidly progressive bilateral DOV for one day. On examination, the patient had no PL in both eyes. Fundus revealed bilateral papillitis (**Fig. 2A**). Contrast-enhanced MRI brain and orbit showed multiple enhancing nodular lesions in bilateral cerebral hemispheres at the grey white matter interface (**Fig. 2B**) along with enhancement of both optic nerves and chiasma. A diagnosis of inflammatory tuberculomas with bilateral AON was made. Additionally, the parents reported two episodes of high-grade fever with self-limiting bilateral paraparesis and bladder and bowel disturbances with LETM on MRI seven months back. A repeated MRI of the spine after recovery showed no significant abnormality. AQP4-Ab and CSF analysis were negative and sputum for acid-fast bacilli ruled out pulmonary tuberculosis.

The patient was treated as a case of NMOSD with tubercular association. ATT along with intravenous methylprednisolone 500 mg/ day was started within 1 day of presentation. After 3 days, vision improved to 2/ 60 in both eyes. Ophthalmoscopy showed mild temporal pallor (**Fig. 2C**). He was started on oral prednisolone 1 mg/ kg/ day for 30 days, which was tapered over the next 4 months. After 6 months, the vision was 6/ 12 in RE and 6/ 9 in LE with persistent mild temporal pallor in both eyes.

## Discussion

NMOSD is an autoimmune astrocytopathy that manifests as bilateral or recurrent ON and LETM. However, other systemic conditions with similar features may co-exist. Early and accurate diagnosis plays an important role since NMO is associated with a poorer prognosis in children [**1**]. Tubercular neuritis is one such condition. The overlapping neurological manifestations make it difficult to discover the primary cause of neuritis. A negative AQP4-Ab assay adds to the diagnostic dilemma [**2**]. Previous studies infer poor visual prognosis for NMOSD associated systemic disorders. However, all such studies have been performed on adults. Our case series showed that the early initiation of systemic steroids in pediatric cases may lead to good visual recovery.

Our first case was initially diagnosed with infectious optic neuropathy. By the time steroids were started, optic atrophy had set in. The second case presented with ON, LETM and intracranial tuberculomas without any signs of pulmonary tuberculosis. Negative CSF analysis ruled out an infectious etiology. Hence, systemic steroids were started promptly. The history of LETM in both cases raised the suspicion of NMOSD.

NMOSD is usually seen in young females, whereas both our cases were males. Extremely rare presentation of NMOSD in children made these cases a bigger diagnostic challenge. The usual picture of involvement of optic nerve preceding spinal inflammation in NMOSD cases was also contradicted by our cases. 

The presence of tuberculomas in case 2 and active cavitary lesions in case 1 clinched the diagnosis of AON in the two cases. The absence of AQP4-Ab in their sera also weakened the diagnosis of NMOSD. Though AQP4-Ab may be a differentiating biomarker between NMOSD and other phenotypically overlapping conditions, 10-40% of patients with NMOSD are found to be seronegative for AQP4-Ab. A high level of clinical suspicion and picking up the minor symptoms may prove vital in such cases.

Multiple studies point towards a common immunological correlation between NMOSD and tuberculosis in adults. A study reported the presence of active TB in NMO cases with controls [**3**]. Another study demonstrated a beneficial effect of ATT on steroid refractory NMO [**4**]. One case report revealed fatal outcome of NMOSD associated with tuberculosis despite active management [**5**]. This is also the only case report with positive AQP4-Ab in TB associated NMOSD. 

## Conclusion

The various disorders that mimic NMOSD are distinct from NMOSD in pathogenesis and treatment. Thus, it is of vital importance for the treating clinician to be vigilant to provide appropriate and timely treatment for better outcomes. The contrasting courses of treatment in the above cases cannot stress more on the importance of early intervention for good visual recovery and near normal final visual acuity.

**Conflict of Interest statement**

The authors state no conflict of interest.

**Informed Consent and Human and Animal Rights statement**

Informed consent has been obtained from all individuals included in this study.

**Authorization for the use of human subjects**

Ethical approval: The research related to human use complies with all the relevant national regulations, institutional policies, is in accordance with the tenets of the Helsinki Declaration, and has been approved by the review board of Command Hospital, Pune, India.

**Acknowledgements**

None.

**Sources of Funding**

None. 

**Disclosures**

None.

**Presentation at a meeting**

NA.
